# Altered Movement Coordination during Functional Reach Tasks in Patients with Chronic Low Back Pain and Its Relationship to Numerical Pain Rating Scores

**DOI:** 10.3390/jimaging10090225

**Published:** 2024-09-12

**Authors:** Susanne M. van der Veen, Christopher R. France, James S. Thomas

**Affiliations:** 1 Department of Physical Therapy, East Carolina University, Greenville, NC 27858, USA; vanderveens24@ecu.edu; 2 Department of Psychology, Ohio University, Athens, OH 45701, USA; france@ohio.edu; 3 Department of Physical Therapy, Virginia Commonwealth University, Richmond, VA 23284, USA

**Keywords:** chronic low back pain, lumbar flexion, kinesiophobia, reaching task

## Abstract

Identifying the effects of pain catastrophizing on movement patterns in people with chronic low back pain (CLBP) has important clinical implications for treatment approaches. Prior research has shown people with CLBP have decreased lumbar-hip ratios during trunk flexion movements, indicating a decrease in the contribution of lumbar flexion relative to hip flexion during trunk flexion. In this study, we aim to explore the relationship between pain catastrophizing and movement patterns during trunk flexion in a CLBP population. Participants with CLBP (N = 98, male = 59, age = 39.1 ± 13.0) completed a virtual reality standardized reaching task that necessitated a progressively larger amount of trunk flexion. Specifically, participants reached for four virtual targets to elicit 15°, 30°, 45°, and 60° trunk flexion in the mid-sagittal plane. Lumbar flexion was derived from the motion data. Self-report measures of numerical pain ratings, kinesiophobia, and pain catastrophizing were obtained. Pain catastrophizing leads to decreased lumbar flexion angles during forward reaching. This effect is greater in females than males.

## 1. Introduction

Whereas low back pain has been associated with reduced lumbar-hip ratios during the forward bend test [1,2,3,4], neither lumbar flexion nor hip flexion has been associated with low back pain when considered in isolation [5,6]. Recently, Peebles et al. (2022) extended this finding by demonstrating that participants with chronic low back pain had reduced lumbar-hip ratios, but not lumbar or hip flexion excursion, in either discrete or cyclic forward bending movement [7]. The fact that the lumbar-hip ratio is reduced in the absence of a reduction in lumbar flexion or an increase in hip flexion suggests that the flexion patterns of the lumbar spine and hip are more variable among subjects with or without back pain. Instead of a reduction or increase in the flexion angles in either joint, the coordination between the flexion movements is affected in patients with back pain. More importantly, if lumbar flexion itself is not reduced among patients with low back, then observed changes in movement coordination may be less a result of the initial cause of their back pain and more a result of learned strategies to avoid pain. In the extreme, this avoidance of movement is called kinesiophobia, and among those with chronic low back pain is associated with impaired recovery [8].

Individuals with acute or subacute low back pain (back pain for less than 3 months) have a less lumbar contribution to trunk motion during the forward bend test compared to controls [2]. Additionally, lumbo-pelvic coordination has been shown to be more synchronized and less variable than that of asymptomatic controls [2]. These alterations could be neuromuscular alterations to avoid painful positions [9] and to protect injured tissues [10]. If the changes in lumbo-pelvic coordination were solely to avoid mechanically-induced pain and to protect damaged tissue, it would be expected that individuals with greater low back pain would limit lumbar flexion more and move more in-phase than people with less pain. In contrast, Shojaei et al. (2020) reported that a subsample of 21 patients with non-chronic low back pain who had low-to-moderate pain showed less lumbar flexion and/or in-phase lumbo-pelvic coordination than 8 matched patients with moderate-to-severe pain [11]. In addition, while pain and disability decreased over a 6-month period, lumbar flexion contribution in the forward bend test and lumbo-pelvic coordination persisted or worsened [11]. This could indicate that avoidance of lumbar flexion and change in coordination is less of a mechanical strategy to avoid acute pain and injury and more of a psychologically-driven adaptation to avoid expected pain and injury, as posited by the fear-avoidance model [12,13].

The fear-avoidance model of low back pain explains why some people with acute musculoskeletal pain go on to develop chronic pain and disability [12,13]. Central to this model, individuals who have catastrophic thoughts about pain (e.g., “Pain is a sign that I am harming my back”), are more likely to experience fear of movement and thoughts of potential re-injury (i.e., kinesiophobia), and hence will engage in behavioral adaptations to avoid or escape situations that they perceive to be associated with such pain. Fear in these studies is defined by a median split on the pain anxiety symptoms scale (56 for females and 59 for males; however, more recent publications have used the Tampa scale of kisesiophobia (TSK > 27) [14,15,16,17]. While the fear-avoidance model posits a generic avoidance of all movements perceived as threatening, we have repeatedly shown that individuals with low back pain and high fear of movement display a particular pattern of motor behavior—they avoid lumbar spine flexion [14,15]. Importantly, individuals with high fear have the available range to flex the lumbar spine, but they choose not to do so. In one study [15], for example, participants with high fear were able to flex the lumbar spine at least 30 degrees when reaching low targets, yet still moved less than those with low fear when reaching targets that required less than 20 degrees of motion. Over time, avoidance of lumbar spine motion increases the risk of re-injury due to the shortening of peri-articular connective tissues and changes in the surrounding muscles [18,19,20].

In the literature, it has been repeatedly reported that females tend to catastrophize pain more than males [21,22,23,24,25]. In line with the fear-avoidance model of low back pain, this higher incidence of pain catastrophizing in females could be a probable cause of increased pain chronification in females [25,26,27]. This difference in males versus females can be explained through social and cultural norms, i.e., males are expected to be stoic, minimizing, and enduring pain, leading to underreporting of pain and pain catastrophizing in males [28]. Where this may affect the care and treatment of pain in a male population, it may help them select more appropriate coping strategies than females [22,29,30]. The fact that females tend to choose less effective coping strategies in addition to already using less lumbar flexion, even without chronic low back pain (CLBP) [31,32], could explain the higher incidence of CLBP in females than males. Besides providing an explanation for the higher incidence of CLBP in females, the link with pain catastrophizing and avoidance of movement, and lumbar flexion in a back pain population, could help us identify better treatment strategies for low back pain, reducing the development of CLBP.

In the present study, we examined pain catastrophizing as potential correlates of the reduction in lumbar flexion observed among individuals with chronic low back pain. We hypothesized that lumbar flexion would be related to catastrophizing. In addition, we posited that these relationships would be stronger for tasks necessitating greater lumbar flexion.

## 2. Materials and Methods

### 2.1. Design

This study utilized an observational design with correlations to examine the relationships between the pain catastrophizing scale (PCS), lumbar flexion angles and lumbar-hip ratio (LHratio) during a standardized reaching task at four heights.

### 2.2. Setting

This study reports findings of a secondary analysis of the virtual immersive gaming to optimize recovery (VIGOR) phase II randomized clinical trial. For more information on the overarching study background and objectives, refer to the prior publication by France & Thomas (2018) [33].

### 2.3. Participants

Participants were recruited through advertisements and flyers posted in the local community and via a combination of electronic, radio, print, and possibly television announcements in the local and surrounding communities. Interested individuals who responded to the recruitment efforts were directed to complete a prescreening survey using REDCap [34], which is a secure online survey and database management application (or a telephone interview, if needed). This pre-screening survey included the main inclusion and exclusion criteria, including a numeric pain rating scale (24 h and 7-day recall), the Roland-Morris Disability Questionnaire, a fear of physical activity question, and medical history related to back pain. Based on their responses, individuals who met the inclusion/exclusion criteria were invited to a full screening session.

During the consent process in a quiet, private room (IRB approval HM20014058), the details of this study were explained to the potential participants. If the potential participant wished to continue, they were asked to read and sign an informed consent document. Those who provided informed consent then completed a series of screening surveys, including a repeat of those completed as part of the pre-screening. Based on their responses to the screening surveys, individuals who continued to meet the inclusion/exclusion criteria then underwent a physical exam by a physical therapist. Participants who remained eligible following the physical exam were formally enrolled in this study.

Participants then proceeded to a baseline assessment, including a series of survey measures and a standardized reaching task. The survey measures included numeric pain rating scales (current, 24 h, and 7-day recall), the Roland-Morris Disability Questionnaire [35], a medication log, and a range of psychological measures (e.g., Tampa Scale for Kinesiophobia [36], Center for Epidemiologic Studies—Depression [37], Pain Catastrophizing Scale [38], and Pain Resilience Scale [39,40]. 

**Figure 1:**
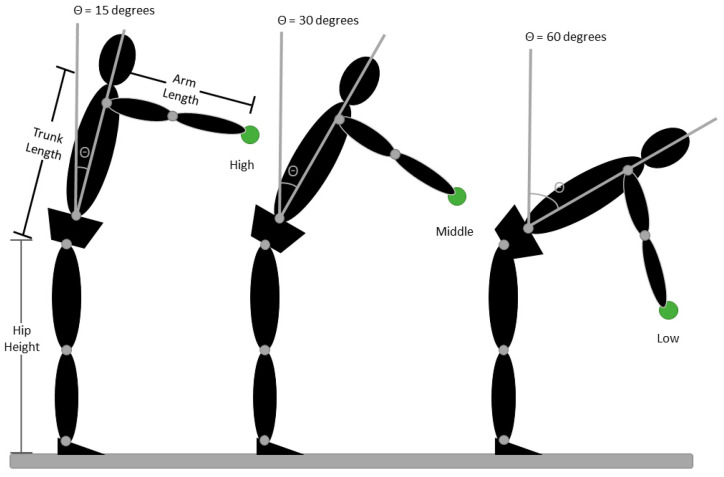
A diagrammatic representation of how target locations were normalized to each subjects’ anthropomorphic characteristics. Target locations were determined for each subject based on their hip height, trunk length, and arm length. The high target was located such that the subject could, in theory, reach the target by flexing the hips 15° with the shoulder flexed to 90° and the elbow extended. The low target could be reached by flexing the hips 60°. Target locations were determined mathematically, and subjects were not actually placed in the positions illustrated.

**Figure 2:**
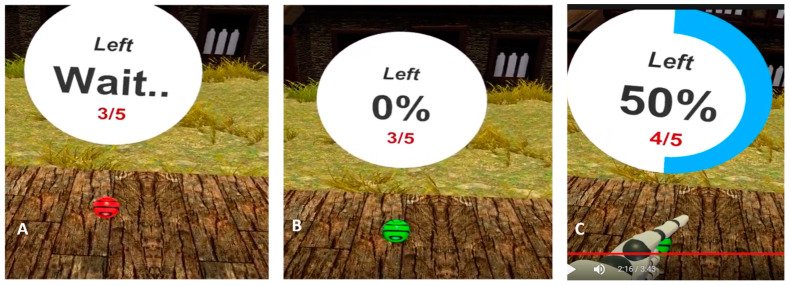
The virtual target was a red-sphere 5 cm in diameter (**A**) that turned green after two seconds to indicate the “go” signal (**B**). Participants touched the sphere with the hand of their avatar, and a white disk 10 cm above the virtual target provided visual feedback to the participant to maintain target contact for two seconds (**C**).

For the standardized reaching task, participants wore a head-mounted display (Vive Pro, HTC, Bellevue, WA, USA) and pointed to virtual targets while the movement of light-reflective marker clusters attached to their head, upper arms, forearms, hands, trunk, pelvis, thighs, shanks, and feet was recorded using a 12-camera Vicon Bonita system. This optoelectric-based kinematic system can track the three-dimensional coordinates of light-reflective marker clusters attached to the participant with a spatial resolution of 0.1 mm. Kinematic data were sampled at 100 Hz. Participants pointed with their hands into 4 virtual targets co-located in the mid-sagittal plane. As shown in Figure 1, target locations are adjusted to participant anthropometrics to allow for comparison of movement patterns across individuals in a task that requires progressive increases in lumbar spine flexion. Specifically, the participant could, in theory, reach the high target by flexing the trunk 15, 30, 45, or 60° with the shoulder flexed 90° and the elbow fully extended (Figure 1) [15]. The virtual target was a red-sphere 5 cm in diameter that turned green after two seconds to indicate the “go” signal. Participants touched the sphere with the hand of their avatar, and a white disk 10 cm above the virtual target provided visual feedback to the participant to maintain target contact for two seconds (see Figure 2). Participants completed 4 trials to each target height with the right hand and then repeated the sequence with the left hand. Lumbar and hip flexion were defined as the change in joint angle during each reach (i.e., the difference between the joint angle at the beginning of the trial before the go signal and the joint angle recorded 100 ms after target contact). These lumbar and hip flexion measures are then used to calculate the lumbar hip ratio (LHratio), $LHratio=\frac{lumbar\;flexion}{hip\;flexion}$ [7]. Consistent with our prior work [15,16,17], expectations of pain and harm were measured. Specifically, for each target height, prior to the first reaching trial, participants rated the level of “expected pain” and “expected harm” using a visual analog scale displayed through the head-mounted display. 

### 2.4. Statistical Analysis

**Figure 3:**
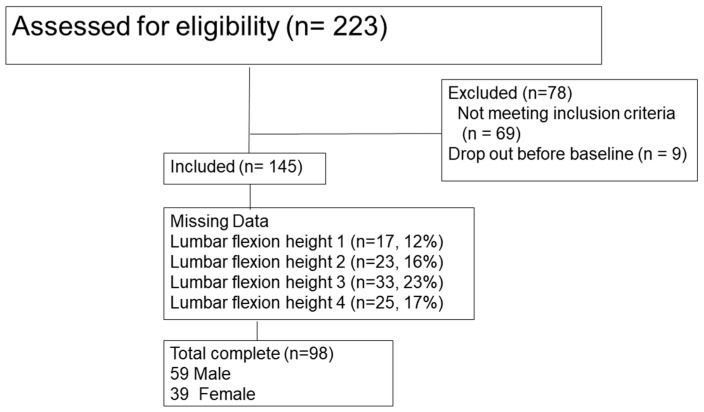
Consort diagram. Total participants assessed for eligibility for the Phase II randomized VIGOR trial, the number of participants excluded due to not meeting inclusion criteria, and the number of participants that dropped out, i.e., that did not show up for the first treatment visit. the number of included participants, with missing lumbar flexion angles due to missing data of the sacrum and/or lumbar marker clusters.

Participant characteristics were summarized using means and standard deviations or frequencies and percentages (see Table 1). Missing data of lumbar flexion angle were excluded from the partial correlation (IBM statistics, SPSS 29), see Figure 3. Partial one-tailed correlations were completed with as variables the PCS and lumbar flexion angles or LHratio for reaching heights 1, 2, 3, and 4, controlled for pain.

Additionally, correlation analyses were followed by separate hierarchical regression for each target location to examine the relationship between Pain Catastrophizing Scale subscale scores (i.e., rumination, magnification, helplessness) with lumbar flexion angles. In these analyses, gender was entered first as an independent variable, followed by the three Pain Catastrophizing Scale subscale scores. Data were assessed for multicollinearity, no values above 2.7 were found [variance inflation factor (VIF) > 10]. Statistical significance was determined using a Benjamini-Hochberg correction for multiple comparisons [41], and reported *p*-values were before correction for multiple comparisons.

## 3. Results

This study included data from 98 participants with CLBP (male = 39, age = 59.06 ± 12.95). See Table 1 for demographics.

### 3.1. Lumbar Flexion

The pain catastrophizing scale (PCS) score showed significant negative correlations with lumbar flexion at height 1 (r = −0.272, *p* < 0.004), height 2 (r = −0.173, *p* = 0.045), height 3 (r = −0.197, *p* = 0.027), and height 4 (r = −0.187, *p* = 0.033). These results indicate that on average, CLBP participants with higher PCS scores use less lumbar flexion during a trunk-bending task. See Table 1.

When separate correlations were completed for male and female CLBP participants, female CLBP participants showed stronger correlations with lumbar flexion angles than males for the different heights. See Table 2. A significant difference was found between male and female correlation coefficients between lumbar flexion to height 1 and PCS (*p* = 0.022) but not at any of the other heights.

Results of the regression analyses indicated that gender was significantly associated with lumbar flexion angle at heights 1 and 2 (with females showing greater lumbar flexion), but not at heights 3 and 4. In addition, while controlling for gender, examination of Pain Catastrophizing Scale subscale scores revealed that higher helplessness scores were associated with less lumbar flexion at all target heights. In contrast, higher magnification scores were associated with greater lumbar flexion at heights 1, 2, and 4, and higher rumination scores were associated with greater lumbar flexion at heights 2, 3, and 4. See Table 3, Table 4, Table 5 and Table 6.

### 3.2. Lumbar Hip Ratio

The pain catastrophizing scale (PCS) score showed no significant correlations with lumbar flexion at any of the four heights. No differences were found between males and females. See Table 7.

## 4. Discussion

The goal of this study was to determine the relationships between lumbar flexion during standardized full body reaching tasks requiring forward bending and pain catastrophizing measures in CLBP participants. Forward bending motions are generally executed by combining lumbar and hip flexion, however, the ratio of flexion in these joints has been shown to be affected by pain and fear of movement in the literature [2,11,14,15]. Since this study included only participants with CLBP and high fear of movement (TSK > 37) we suspected that lumbar flexion would show associations with measures of pain catastrophizing. This study reinforces that movement patterns in forward bending and specifically lumbar flexion angles are affected by pain catastrophizing.

On average, lumbar flexion declines in CLBP participants with kinesiophobia when they are asked to bend over further, see Table 7. The ratio between lumbar and hip flexion (LHratio) represents less lumbar and more hip contribution to trunk flexion movement patterns. Lumbar flexion seems to plateau around 30 degrees flexion in the third reaching task (45 degrees trunk flexion). This means that when participants are asked to touch a target that elicits 60 degrees of trunk flexion, participants still use about 30 degrees of lumbar flexion, but mainly increase hip flexion. To illustrate lumbar flexion in a healthy population has been reported between 40 and 73 degrees [42], of which about 48 degrees were used when picking up an object from the ground (bending at the waist) [43]. In functional range of motion tests (flexion-extension, lateral bending, rotation), the range of motion is reduced compared to healthy controls [44]. However, flexion motion was reduced in LBP participants, and the variability was high, so no specific indication of limitation in lumbar flexion can be made [44]. So, the overarching message is, people with LBP have reduced lumbar flexion contribution to trunk flexion, no direct comparison of our results can be made with existing research as virtual reality reaching tasks have shown different dynamics compared to real-world reaching tasks [45]. 

Based on the fear-avoidance model in low back pain, we hypothesized that lumbar flexion angles would be reduced in patients with CLBP, who have high fear and catastrophizing characteristics. In contrast, we expected no correlation in LHratio within a participant population with pain and kinesiophobia, as LHratio discriminated between CLBP participants and no pain controls, however, is likely not discriminatory of pain catastrophizing. These hypotheses were based on the findings of multiple studies showing that individuals with low back pain and high fear avoid lumbar spine flexion [14,15,16]. Importantly, individuals with high fear have the available range to flex the lumbar spine, but they choose not to do so. Additionally, even when the back pain decreases, lumbar flexion contribution and lumbar-pelvic coordination do not normalize [11]. In line with these previous findings, we found that lumbar flexion angles were significantly reduced in CLBP participants with pain catastrophizing. As expected, this effect is stronger in females than males (see Table 2). In the previous literature we have shown males and females have vastly different movement patterns, i.e., men tend to have higher lumbar flexion contributions to trunk flexion [31]. In this study, we did not see these differences in lumbar hip ratios between males and females. However, we have a CLBP population with a high fear of movement, females in general are more likely to pain catastrophizing which is linked to pain chronification [27]. We suggest future research to investigate the effect of low and high fear and gender on lumbar flexion angles and trunk flexion contributions in participants with CLBP.

The lumbar contribution to trunk flexion in this VR-based whole-body reaching task is inversely related to the level of pain catastrophizing even when controlling for current pain levels. This relationship was significant for all four target heights even though reaching the high targets necessitated trivial amounts of lumbar flexion. Perhaps, of greater importance is this cohort all had high levels of kinesiophobia (i.e., >37 on the TSK) but the pain catastrophizing scale revealed the powerful role of fear cognitions in expressed motor behavior in chronic back pain and this must be addressed in rehabilitation strategies. More specifically, regression analysis showed that the helplessness subscale of the PCS showed significant association with decreased lumbar flexion angles at all four target heights, whereas the rumination and magnification subscales showed positive associations with greater lumbar flexion at three of the four target heights. These findings indicate that a sense of helplessness is driving the inverse correlations observed between the PCS total scale score and decreased lumbar flexion at all target heights. Because the helplessness subscale of the PCS reflects a respondents’ perceived inability to cope with their back pain and a sense that their suffering will continue indefinitely due to an inability to change the situation, rehabilitation of those with chronic low back pain may benefit particularly from greater efforts to enhance pain coping skills that offer hope for restoration of function.

As mentioned in the prior paragraph, the fact that all patients with CLBP in this study had kinesiophobia (TSK < 27) likely caused a ceiling effect in our results, which is a limitation of this study. In future studies, associations should be explored within patients with CLBP who have a wide range of kinesiophobia and pain scores to provide a full view of how these relationships can inform clinicians about movement patterns, pain, and fear of pain.

## 5. Conclusions

The lumbar flexion reduction is significantly related to increased pain catastrophizing in CLBP participants. This relationship between pain catastrophizing and lumbar flexion angle, i.e., lumbar movement magnitude, is greater in females than in males. However, although the literature indicates that LHratio is a good distinguisher between patients with back pain and healthy controls, LHratio did not show a relationship with kinesiophobia or fear of movement.

## Figures and Tables

**Table 1:** Demographics, Tampa kinesiophobia (TSK), pain resilience scale (PRS), Pain Catastrophizing scale (PCS), numeric pain rating scale (NRS).

Measure	Mean ± SDV
Gender	39 Female/59 Male
Age (years)	39 ± 13
Height (cm)	169 ± 10
Weight (kg)	86.9 ± 25.4
TSK	43.1 ± 5.9
PRS	34.1 ± 11.0
PCS	20.3 ± 11.0
NRS now	4.7 ± 2.0
NRS 24 h	5.1 ± 2.0
NRS 7-day	5.6 ± 1.9

**Table 2:** Correlation table for pain catastrophizing scale (PCS) and lumbar flexion angles for all participants, and male and female participants separate, normalized for pain (NRS now).

PCS	All	Male	Female
Lumbar flexion angle Height 1	−0.272 **	−0.113 *	−0.496 **
Lumbar flexion angle Height 2	−0.173 **	−0.72	−0.290 **
Lumbar flexion angle Height 3	−0.197 **	−0.165 *	−0.226 **
Lumbar flexion angle Height 4	−0.187 **	−0.135 *	−0.252 **

Significance of a correlation is represented with an * for a *p* < 0.05 and ** for a *p* < 0.001.

**Table 3:** Hierarchical regression results for lumbar flexion angles at reach height 1. Pain Catastrophizing scale (PCS).

Height 1	Model 1				Model 2			
	B	SE	t	*p*-Value	B	SE	t	*p*-Value
R^2^	0.002				0.069			
(Constant)	12.29	0.353	34.87	<0.001	13.59	0.63	21.46	<0.001
Gender	−3.21	0.57	−5.64	<0.001	−3.24	0.56	−5.78	<0.001
PCS rumination					−0.02	0.10	−0.23	0.820
PCS magnification					0.483	0.13	3.65	<0.001
PCS helpless					−4.77	0.09	−4.77	<0.001

**Table 4:** Hierarchical regression results for lumbar flexion angles at reach height 2. Pain Catastrophizing scale (PCS).

Height 2	Model 1				Model 2			
	B	SE	t	*p*-Value	B	SE	t	*p*-Value
R^2^	0.008				0.111			
(Constant)	22.22	0.55	40.47	<0.001	25.66	0.97	26.40	<0.001
Gender	−2.13	0.89	−239	0.017	−2.05	0.85	−2.42	0.016
PCS rumination					0.39	0.14	2.75	0.006
PCS magnification					0.58	0.21	2.82	0.005
PCS helpless					−1.13	0.13	−8.65	<0.001

**Table 5:** Hierarchical regression results for lumbar flexion angles at reach height 3. Pain Catastrophizing scale (PCS).

Height 3	Model 1				Model 2			
	B	SE	t	*p*-Value	B	SE	t	*p*-Value
R^2^	0.000				0.040			
(Constant)	28.47	0.76	37.65	<0.001	28.88	1.21	22.01	<0.001
Gender	−0.69	1.20	−0.58	0.565	−0.60	1.18	−0.51	0.611
PCS rumination					0.70	0.21	3.43	<0.001
PCS magnification					0.43	0.29	1.50	0.133
PCS helpless					−0.95	0.18	−5.18	<0.001

**Table 6:** Hierarchical regression results for lumbar flexion angles at reach height 4. Pain Catastrophizing scale (PCS).

Height 4	Model 1				Model 2			
	B	SE	t	*p*-Value	B	SE	t	*p*-Value
R^2^	0.002				0.069			
(Constant)	31.02	0.77	40.48	<0.001	34.60	1.33	26.06	<0.001
Gender	1.40	1.27	1.11	0.269	1.97	1.23	1.61	0.109
PCS rumination					0.43	0.21	2.07	0.038
PCS magnification					0.85	0.30	2.82	0.005
PCS helpless					−1.34	0.19	−7.02	<0.001

**Table 7:** Outcome measures per movement height. Lumbar Hip Ratio (LHratio). Lumbar and hip flexion angles are reported in degrees, pain and harm expectancies are returned scores on visual analog scales (0–100) of the participants’ expectation of pain and harm while moving to the four target heights.

Measure	Height 1	Height 2	Height 3	Height 4	F	*p*
LHratio	0.97 ± 0.91	0.86 ± 0.73	0.64 ± 0.49	0.57 ± 0.37	14.390	<0.001
Lumbar flexion	11.1 ± 7.9	21.4 ± 11.8	28.2 ± 15.3	31.5 ± 16.6	75.315	<0.001
Hip flexion	15.4 ± 10.8	33.8 ± 16.6	49.9 ± 16.7	60.5 ± 17.9	247.025	<0.001
Pain expectancy	27 ± 21	33 ± 23	41 ± 24	43 ± 27	22.833	<0.001
Harm expectancy	25 ± 27	31 ± 28	35 ± 29	40 ± 30	11.822	<0.001

## Data Availability

Deidentified participant data is available upon email request to James Thomas (jthomas32@vcu.edu) after publication with assigned data access agreement and the proposed use of the data are approved.
